# Case Report: Association of a Variant of Unknown Significance in the *FIG4* Gene With Frontotemporal Dementia and Slowly Progressing Motoneuron Disease: A Case Report Depicting Common Challenges in Clinical and Genetic Diagnostics of Rare Neuropsychiatric and Neurologic Disorders

**DOI:** 10.3389/fnins.2020.559670

**Published:** 2020-12-22

**Authors:** Caroline Gertrud Bergner, Christiane Michaela Neuhofer, Claudia Funke, Saskia Biskup, Philipp von Gottberg, Claudia Bartels, Jan Christoph Koch, Katrin Radenbach

**Affiliations:** ^1^Department of Neurology, University Medical Center Göttingen, Göttingen, Germany; ^2^Department of Neuropathology, University Medical Center Göttingen, Göttingen, Germany; ^3^Department of Genetics, University Medical Center Göttingen, Göttingen, Germany; ^4^CeGAT, Tübingen, Germany; ^5^Department of Neuroradiology, University Medical Center Göttingen, Göttingen, Germany; ^6^Department of Psychiatry and Psychotherapy, University Medical Center Göttingen, Göttingen, Germany

**Keywords:** amyotrophic lateral sclerosis, dementia, *FIG4*, neurodegeneration, genetics

## Abstract

**Background:**

Modern genetics have in many ways revolutionized clinical routine and have, for instance, shown that formerly distinct disease entities relate to common pathogenic mutations. One such example is the connection between dementia and amyotrophic lateral sclerosis (ALS) in a continuous disease spectrum affirmed by the discovery of shared mutations.

**Case Report:**

We describe a new variant in the *FIG4* gene in a patient with slowly progressing frontotemporal dementia (FTD) and probable primary lateral sclerosis (PLS). The patient initially showed depressive symptoms and global cognitive deficits. Severe difficulties with language and hallucinations became clearer as the disease progressed. Nuclear medicine imaging and cerebrospinal fluid (CSF) biomarkers were not specific for defined categories of dementia, but neuropsychological testing and clinical features finally led to an allocation of the syndrome to the non-fluent variant of primary progressive aphasia (nfv PPA). Because of increasing limb weakness and bulbar symptoms, motoneuron disease in the form of PLS was diagnosed, strongly supported by elevated CSF neurofilament and electrophysiologic assessments. The detected variant in the *FIG4* gene is described as pathogenic or likely pathogenic in common databases and reported once in the literature. While the phenotype of our patient fits the description of *FIG4*-associated disease in literature, we consider the present variant as VUS in this case.

**Conclusion:**

We describe a variant in the *FIG4* gene in a patient with slowly progressing FTD and PLS. Mutations in the *FIG4* gene have been associated with ALS and PLS; however, this exact mutation was not reported in ALS or PLS patients before. The case illustrates generic diagnostic challenges in patients presenting with genetic variants that offer an explanation for otherwise uncommon symptom combinations but yet are of unknown significance.

## Introduction

Amyotrophic lateral sclerosis (ALS) is the most frequent adult-onset motoneuron disease (Logroscino et al., 2018). Up to 10% of ALS cases have a familial background with mostly dominant inheritance. Until now, more than 25 genes have been associated with ALS that account for approximately 70% of all familial cases. Mutations of these genes, however, are also identified in approximately 15% of non-familial cases, and there is accumulating evidence for a strong genetic contribution in sporadic ALS (Turner et al., 2013).

Heterozygous pathogenic variants in the *FIG4* gene were first described in familial and sporadic ALS and primary lateral sclerosis (PLS) in 2009 and defined as ALS11 (Chow et al., 2009). Until now, only a few clinical reports of patients with ALS11 exist (Osmanovic et al., 2017). We report the case of a 61-year-old Caucasian patient with slowly progressing motoneuron disease and frontotemporal dementia (FTD) possibly associated with a heterozygous *FIG4* mutation. This mutation so far has not been described in ALS or PLS patients.

## Case Report

A 61-year-old female patient initially presented to the psychiatric memory clinic in August 2014. She attended school for 9 years and had been working as an untrained shop assistant in a bakery afterward. According to the patient’s self-report, she had been suffering from recurrent depressive episodes since 2011. Additional cognitive decline developed in 2012. Comprehensive neuropsychological testing was performed and revealed severe and global deficits affecting all major cognitive domains (memory, visuoconstructional and executive functions, language, and attention, including psychomotor slowing). Together with impaired activities of daily living, general *International Classification of Diseases, 10th Revision* criteria for dementia were fulfilled. At several follow-ups, the patient repeatedly mentioned a subjective progression of cognitive deficits. However, in neuropsychological screenings and six comprehensive neuropsychological testings performed since first presentation, the patient presented stable deficits over 5 years, and substantial further decline was found only on the last visit in 2020. Since 2015, the patient experienced increasing acoustic, optic, and tactile hallucinations, and delusional parasitosis led to wounding scratching behavior. IBZM-SPECT (^123^I-iodobenzamide, 2015), amyloid–positron emission tomography (PET) (^18^F-florbetaben, 2016), DaT scan (^123^I-FP-CIT, 2015), and ^18^F-fluorodeoxyglucose-PET (2019) were performed yielding normal results.

**FIGURE 1:**
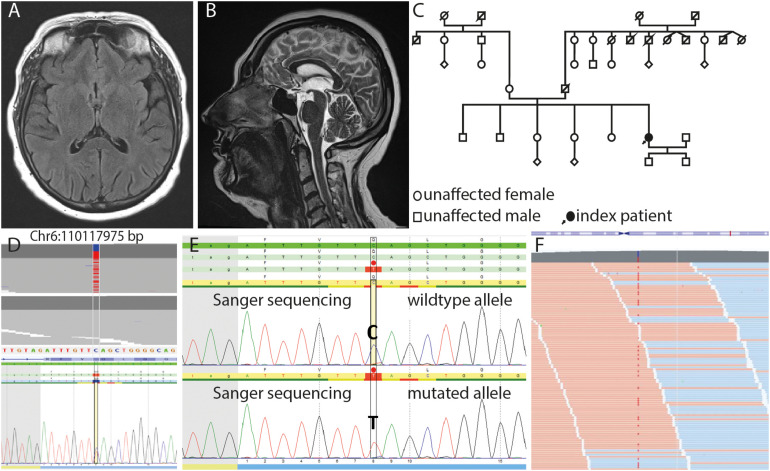
Axial FLAIR **(A)** and sagittal T2w **(B)** images show no morphologic features of neurodegeneration or structural damage to the brain. **(C)** Pedigree of the family. Note that information on clinical symptoms of the patients’ relatives was available only until her age of 20 years. **(D)** JSI-Sanger data with the heterozygous variant in *FIG4* compared to in-house next-generation sequencing (NGS) data, showing this variant in heterozygous state (upper part) and randomized in-house reference pool, which shows the absence of this variant in wildtype (lower part). **(E)** DNA sequence of the wild-type and mutated allele containing the substitution c.2467 C > T, which leads to the nonsense variant p.Q823* on protein level (red dot) in FIG4. **(F)** Mapped sequencing reads show the variant on forward (orange) and reverse (blue) strand in approximately 50% of the reads, which is expected for heterozygosity with a sequencing depth (coverage) of more than 500 reads per base.

While the patient initially presented with mildly slurred speech and reduced prosody, she suffered from progressive dysarthria, apraxia of speech, and increasing difficulties in swallowing in the further course and unintentionally lost 15 kg weight. In addition, she complained about deteriorating limb weakness. Magnetic resonance imaging (MRI) of the cerebrum showed no abnormalities (Figures 1A,B). MRI of the spine showed mild cervical spine degeneration and lateral thoracic spinal canal stenosis with initial myelon deformation, but no myelon compression and edema, respectively. The patient underwent thoracic laminectomy in 2016. However, limb weakness continuously worsened over the next years, and the patient is now increasingly restricted in walking ability.

Neurologic examination revealed pseudobulbar palsy, distal muscular weakness without fasciculations but with slightly increased muscle tone, and clearly increased tendon reflexes including bilateral positive Babinski signs, more severely affecting lower than upper limbs. Electromyography of muscles of all four limbs did not show any indication of active denervation (no fibrillations or positive sharp waves), but signs of mild chronic denervation, i.e., increased motor unit potential amplitudes and reduced recruitment. Neurographies revealed pure axonal motor neuropathy of the right peroneal and tibial nerve and a small reduction of the amplitudes of the right sural nerve. No signs of demyelination were found. Transcranial magnet stimulation revealed a prolonged central conduction time to the left leg. Sonography of the tongue did not show any fasciculations.

Extensive cerebrospinal fluid (CSF) analysis did not show indication of inflammatory or paraneoplastic disorder, and neuronal destruction markers including beta-amyloid, tau, and phosphotau were within normal range. However, neurofilament heavy chain in CSF was significantly elevated (NfH 1137 pg/mL), strongly supporting the diagnosis of motoneuron disease. Taken together, clinical evidence suggests progressive upper motoneuron dysfunction over >3 years in the bulbar, cervical, and lumbar region, while no sensory symptoms and no signs of active lower motoneuron degeneration could be found. According to the recently published consensus criteria, the diagnosis of probable PLS must thus be made (Turner et al., 2020). A critical role of the cervical spinal canal stenosis is unlikely as no signs of myelon compression were detectable on MRI scans, and degeneration of the upper motoneuron was also clearly noticed in the bulbar region.

No neurologic disease was reported in the patients’ family, although only incomplete information was available (Figure 1C). Because of the unusual presentation and suspicion of an underlying hereditary neurodegenerative syndrome, the patient underwent genetic testing with targeted next-generation sequencing panel diagnostics. Heterozygosity for the nonsense-mutation c.2467C>T, p.Q823^∗^ in the *FIG4* gene was detected (Figures 1D–F). The variant is listed as pathogenic for AR CMT4J in the HGMD^®^ database (accession number CM115114) and as likely pathogenic, pathogenic, or VUS in ClinVar (variation ID 447336). The variant is found once heterozygous in the ExAC browser (1/121394 alleles) and seven times heterozygous in gnomAD (7/282818 alleles). The mutation was observed in a screening study in CMT patients by Nicholson et al. (2011); however, a detailed phenotype and whether or not the patient carried biallelic *FIG4*-mutations were not reported.

## Discussion

We describe the case of a patient with probable PLS and dementia, possibly associated with the heterozygous variant Gln823^∗^ in the *FIG4* gene. This variant so far has not been described in patients with ALS or PLS.

The *FIG4* gene codes for a phosphoinositide (3,5) P_2_ phosphatase that regulates levels of phosphoinositide (3,5) P_2_ in vacuolar membranes (Rudge et al., 2004). Pathogenic variants in *FIG4* are thought to be associated with several distinct phenotypes—autosomal recessive inherited CMT (CMT4J, phenotype MIM number 611228), autosomal recessive inherited Yunis-Varon syndrome (phenotype MIM number 216340), and autosomal dominant inherited ALS (ALS11, phenotype MIM number 612577). Another nonsense mutation in *FIG4*, p.R183^∗^, has previously been shown to be causative of a CMT phenotype when found biallelic with another missense variant [I41T, (Chow et al., 2007)] and at the same time a risk factor for ALS11 when heterozygous (Chow et al., 2009). The observation of clinically unaffected carriers was attributed by Chow et al. to late onset of symptoms (carriers might yet develop symptoms) or incomplete penetrance. This assumption would also explain the occurrence of the p.R183^∗^ mutation in a healthy population; the variant is found three times heterozygous in ExAC (3/121218 alleles) and seven times in gnomAD (7/245708). These frequencies are similar to the ones observed for the p.Q823^∗^ variant found in our patient.

The clinical course of our patient is remarkable as it shows an initial predominance of cognitive and affective symptoms and a very prolonged disease duration. Eight years after disease onset, she shows only moderate motoric affection and is ambulatory without aid for more than 500 m. The lack of acute denervation in electromyography and absence of fasciculations fit with the observed very slow progression of motor symptoms and the probable PLS. Elevated neurofilament heavy-chain levels in CSF strongly support the diagnosis of PLS or another ALS spectrum disorder. FIG4 protein was found to coaggregate in TDP-43 and in alpha-synuclein–positive inclusions (Kon et al., 2014), thus underlining the putative role in inducing pathological protein aggregation in different neurodegenerative disorders. In our patient, no signs of beta-amyloid deposition or parkinsonism could be found.

The patient underwent extended neuropsychological testings that covered comprehensively all major cognitive domains. For a differential diagnosis of dementia, early Alzheimer disease and Lewy body dementia were considered. However, in the absence of confirmative imaging or CSF biomarkers and with a stable disease course over 5 years, these diagnoses seem unlikely. Instead, following the clinical phenotype and results of our neuropsychological testings, the syndrome of the patient matches the diagnostic criteria of FTD, with the clinical characteristics of the non-fluent variant primary progressive aphasia (PPA) [fulfilled features: apraxia of speech, impaired comprehension of syntactically complex sentences, spared single-word comprehension, and spared object knowledge (Gorno-Tempini et al., 2011)]. Additionally, she displayed mild behavioral symptoms (apathy and lack of empathy), as confirmed by the Frontal Behavioral Inventory. FTD is a slowly progressing neurodegenerative disorder, known to be commonly associated with ALS (Strong, 2008). Recent data suggest that in the PPA subtype of FTD ALS might occur in frequencies comparable to that found in the behavioral variant of the disease (Tan et al., 2019). Hallucinations are not typical in ALS but have been reported (Zucchi et al., 2019).

Recent clinicopathological and genetic discoveries in the field of ALS have motivated a repositioning of the disease within a broadened pathogenetic and clinical spectrum including central nervous system functions outside the motor system and softened distinctions to other neurodegenerative diseases (Strong, 2008; Pradat et al., 2009). Moreover, the concept of a unified disease entity of ALS is questioned by the now revealed diversity of associated mutations and histopathological heterogeneity (Turner et al., 2013).

In summary, based on aforementioned genetic findings and taking into consideration the patient’s characteristic phenotype, which is mostly incongruous with other neurodegenerative diseases but fits well into such a novel delineation of ALS phenotypes, we consider the here described c.2467C>T/p.Q823^∗^ variant as a variant of unknown significance, but possibly causative for our patients’ symptoms. However, further functional and clinical studies in years to come are necessary and will hopefully allow to pinpoint the pathologic significance of this variant more precisely. Our patient exemplifies how nowadays genetics may be helpful to better allocate patients with uncommon symptom combinations to a disease entity. At the same time, the case illustrates that taking into account unknown clinical significance of a specific variant, genetic results should only be one, although important, piece of the diagnostic puzzle. Remaining diagnostic uncertainty in addition to the broad clinical spectrum of disease courses associated with a given ALS gene turns prognostic estimates and genetic counseling into a challenge. Careful clinical and genetic reassessment and repeated prognostic counseling of the patient and potentially affected relatives in light of further genetic and epidemiologic insights yet to come are mandatory.

## Data Availability Statement

The datasets presented in this article are not readily available because genetic data refer to an individual patient and therefore are considered very personal. Requests to access the datasets should be directed to the corresponding author.

## Ethics Statement

Written informed consent was obtained from the individual(s) for the publication of any potentially identifiable images or data included in this article.

## Author Contributions

CGB, CB, JK, and KR examined the patient clinically. PG performed and analyzed neuroradiologic imaging studies. CN, CF, and SB performed genetic analyses. CGB, CN, CB, JK, and KR wrote the manuscript. JK and KR contributed equally as senior authors. All authors contributed to the article and approved the submitted version.

## Conflict of Interest

The authors declare that the research was conducted in the absence of any commercial or financial relationships that could be construed as a potential conflict of interest.
